# Neutrophil/Lymphocyte Ratio as Predictor of Anastomotic Leak after Gastric Cancer Surgery

**DOI:** 10.3390/diagnostics10100799

**Published:** 2020-10-09

**Authors:** Dumitru Radulescu, Vlad Dumitru Baleanu, Vlad Padureanu, Patricia Mihaela Radulescu, Silviu Bordu, Stefan Patrascu, Bogdan Socea, Nicolae Bacalbasa, Marin Valeriu Surlin, Ion Georgescu, Eugen Florin Georgescu

**Affiliations:** 1 General Surgery Department, University of Medicine and Pharmacy of Craiova, 200349 Craiova, Romania; dr_radulescu_dumitru@yahoo.com (D.R.); drbaleanu@yahoo.com (V.D.B.); vsurlin@gmail.com (M.V.S.); ion_georgescu@yahoo.com (I.G.); eugenyok@yahoo.com (E.F.G.); 2 Internal Medicine Department, County Hospital of Craiova, University of Medicine and Pharmacy of Craiova, 200349 Craiova, Romania; vldpadureanu@yahoo.com; 3 “Victor Babes” Clinical Hospital of Infectious Diseases and Pneumophtisiology Craiova, University of Medicine and Pharmacy of Craiova, 200349 Craiova, Romania; paty_miha@yahoo.com; 4 General Surgery Department, “Carol Davila” University of Medicine and Pharmacy, 021659 Bucharest, Romania; 5 “Dr. Ion Cantacuzino” Hospital, Gynecology Department, “Carol Davila” University of Medicine and Pharmacy, 020457 Bucharest, Romania; nicolaebacalbasa@gmail.com

**Keywords:** gastric cancer, anastomotic leakage, predictor, NLR

## Abstract

Introduction. Neutrophil/lymphocyte ratio (NLR) is known as a prognostic for the outcome of the patients with gastric cancer. As no definite risk marker for anastomotic leakage after gastric resection was identified, we investigated the possible role of NLR. Methods. Peripheral blood count for neutrophils and lymphocytes was done at the patient’s admission. We retrospectively evaluated 204 gastric cancer patients, who underwent gastric resection, comparing the values of NLR between the group of patients with anastomotic leakage and those without complications. Results. Using the ROC curve, we found the cutoff value of NLR, which permitted the comparison of the group with low NLR, presenting increased NLR. The cutoff value for NLR was 3.54. Between the two groups, we could observe statistically significant differences in developing fistula (*p* < 0.01) and complications leading to death (*p* < 0.025). The odds ratio for patients with NLR greater than 3.54 to develop anastomotic leak was 17.62, compared to those with lower NLR. Conclusion. Peripheral blood NLR proved to be a predictor for anastomotic leakage.

## 1. Introduction

Anastomotic leakage after gastric resections for malignant tumors represents an awful outcome, with high mortality rate, raising the risks of local recurrence and worsening the overall prognosis. The rate of anastomotic gastric dehiscence is between 2.7–12.3% [1], as it is a source of high morbidity and a mortality rate that can tend to 60% [2,3].

The high negative prognostic is due to the consequent peritonitis or mediastinitis, leading to the occurrence of Systemic Inflammatory Response Syndrome (SIRS) and sepsis. Most of the time, the contents discharged through the anastomotic dehiscence are not immediately externalized on the drainage tube, creating a localized collection. This happens even if they are partially externalized, maintaining sepsis. Knowing that reducing the number of drainage tubes leads to reduced hospitalization [4], the need for predictive markers of the risk of anastomotic fistula is becoming greater, in order to use, when necessary, more drainage tubes in patients at risk to successfully treat fistulas.

It is well established that SIRS is associated with changes in white blood cells in the circulation, particularly the presence of neutrophils with relative lymphocytopenia [5]. In general, blood neutrophils increase with the progression of inflammatory disease.

Lymphocytes reflect a patient’s immune status and generally decrease as the inflammatory disease progresses, but this decrease is relatively delayed and may not reflect well the progression of the disease [6].

Recently, studies have shown that NLR is more reliable when predicting patient survival than the number of neutrophils or lymphocytes studied separately [7].

Neutrophils are involved in the healing process of a digestive anastomosis, being attracted to the ischemic areas where they release inflammatory mediators, proteolysis’ enzymes, and free radicals [8,9]. On the other hand, lymphocytes are involved in decreasing inflammation and healing processes [9,10].

Anastomotic fistula treatment involves prolongation of hospitalization and sometimes surgical re-interventions, which are burdened with complications that can lead to increased mortality.

The existence of recognized predictive factors for the healing of the digestive anastomosis can lead to the particularization of the preoperative measures and to choosing a surgical technique, which will lower the incidence of the fistula, and in case of their formation, will lead to the improvement of the management by early diagnosis and intervention.

The neutrophil-lymphocyte ratio in cancer patients has been recently cited as a global predictor of mortality and chemo-sensibility [11,12,13].

A commonly marker of systemic inflammatory status is the neutrophil-lymphocyte ratio (NLR), which is a simple, easily calculable marker, obtained from the absolute numbers of neutrophils and lymphocytes, from the complete blood counting of the figured elements of peripheral blood. NLR has recently been established as a good indicator of systemic inflammatory status in the general population [14]. NLR is proven to have better indicator qualities for systemic inflammation than leukocytes or neutrophils alone [15].

Starting from these data, the study aimed to investigate the role of NLR as a predictor of systemic inflammatory status, as well as the existence of a possible relationship with anastomotic fistula in gastric surgery. Regarding these facts, we wanted to study whether the assumption that NLR values can play a defining role in healing or, conversely, the unfavorable evolution of a digestive anastomosis is real. The inflammatory systemic response is part of the whole healing process, so prolonging it in the context of pre-existing inflammation may increase risks of developing anastomotic leakage.

## 2. Materials and Methods

This study was conducted under the approval of the local ethics council of the County Emergency Clinical Hospital of Craiova (number 2537 from 10 December 2012) and included patients admitted to the Surgery Clinic I, whose diagnosis was gastric cancer. Charts of 204 patients who were treated by a surgical procedure and who have at least one anastomosis with the small bowel were prospectively analyzed in a period of time from January 2013 to December 2019. All patients were operated on by an open approach. We analyzed demographical data (sex, age), histological type of the tumor, staging, the type of surgical intervention, white blood count, platelets, serum proteins, and the neutrophil/lymphocyte ratio.

In our study, neutrophils and lymphocytes were counted from peripheral blood samples at the moment of admission to hospital.

### 2.1. Inclusion and Exclusion Criteria

Patients included in our study were patients with gastric adenocarcinoma, histopathologically confirmed, from January 2013 to December 2019. They had a preoperative peripheral blood count, before performing any treatment, rehydration, and electrolytic or protein status equilibration. Patients who completed the scheme of preoperative chemotherapy, radiotherapy, or both, were also included in the study.

The exclusion criteria were: patients operated on in emergency conditions, those who presented secondary disseminations, patients presenting concomitant malignancies, patients on chronic systemic anti-inflammatory treatment, patients who discontinued the oncological scheme, and patients with evidence of systemic inflammation and personal history of autoimmune conditions.

### 2.2. Study Design

We performed a peripheral blood cells counting at the hospitalization moment and we calculated the results by making the ratio between the percentages of neutrophils and lymphocytes.

Numerical data were presented as absolute numbers or relative frequencies (percentage) for categorical variables.

We expressed results as values of medians with standard deviation for continuous quantitative variables.

### 2.3. Statistical Analysis

Data analysis was done using SPSS version 20 using descriptive statistics of the studied population.

The optimal value resulted from analyzing the characteristic ROC curve, fixing a normal maximum limit in order to obtain results with high specificity and sensitivity in predicting the healing of the anastomosis with the small bowel.

We performed the independent t test in order to determine whether there is any relationship between the appearance of the anastomotic leakage and the NLR.

Data interpretation was considered as statistically significant results if the *p*-values were less than 0.05, with a 95% confidence interval.

Univariate statistical analysis was performed using hypothesis confirmation tests, the chi-square test for qualitative variables, and the Student t test for comparing quantitative variables with homogeneity of variations in the Levene test.

## 3. Results

**Figure 1:**
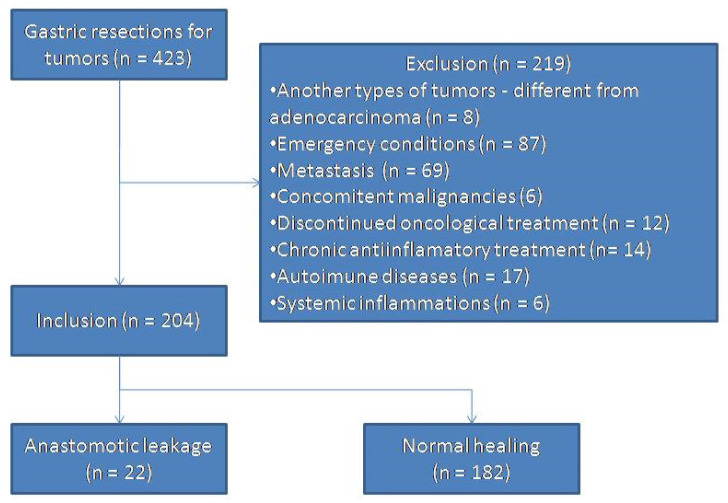
Flow chart of patient selection (NLR = neutrophil-lymphocyte ratio).

A number of 204 patients were included in the study (Figure 1).

The mean age of the patients included in study was 67.75 ± 9.73 years (Table 1), with extremes of 32 and, respectively, 91 years, and a sex ratio M:W of 1.61(61.8% men). The presence of a positive family history of cancer was 7.8%. Extreme values of peripheral blood count for different figured elements can also be seen in Table 1.

Following the diagnosis, tumor staging was as follows: 11.7% stage I, 31.8% stage II, 46% stage III, and 10.2% stage IV (Table 2).

Since we noticed that the NLR value increases in direct proportion to the tumor stage, we used the one-way analysis of variance (ANOVA) test to prove the relationship between NLR and stage. This test has demonstrated that NLR and Tumor Nodules Metastases (TNM) status are interdependent parameters (*p* = 0.020). NLR ranged from 1.49 to 9.75. The mean calculated value was 2.71 ± 1.16.

We found a relationship between the NLR value and the presence of anastomotic leakage, so we were able to divide the patients into two groups: (1) those who developed anastomotic leakage, and (2) those with good postoperative outcome through complete anastomosis healing.

We found a statistically significant difference in the value of NLR between the group of patients with anastomotic fistula and the one without fistula using the t test (*p* = 0.001).

**Figure 2:**
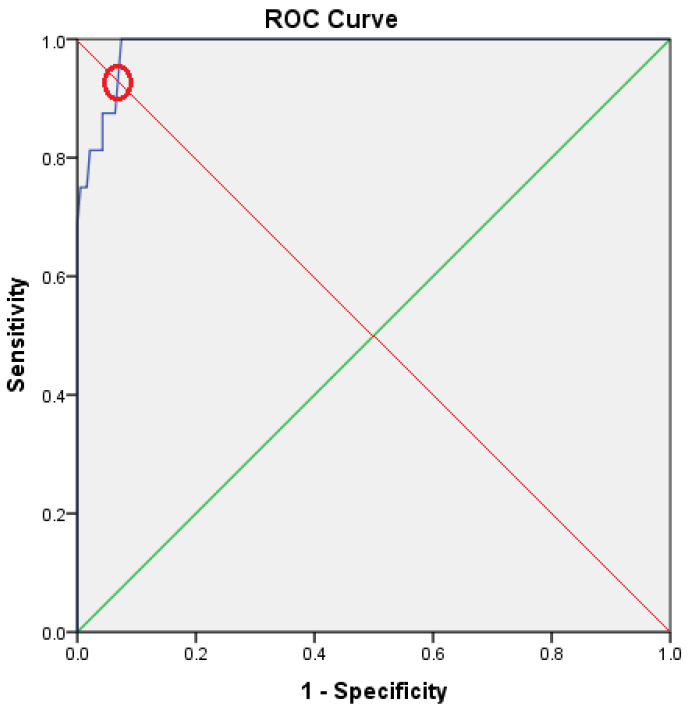
Receiver Operating Characteristic curve.

Among all analyzed parameters, NLR, a marker of inflammation, proved to be the best predictor for anastomotic leak, with a surface over the ROC curve (Receiver Operating Characteristic curve) of 0.987 [0.945–0.992] (*p* < 0.001); (Figure 2).

The value of 3.54 was found as the best cutoff value for NLR, presenting a specificity of 93.6% and 87.0% sensitivity. The subjects included in the study were, in consequence, split into two groups according to theirpreoperative NLR value (lower than 3.54 vs. higher than or equal to 3.54), One can find the variables for the low NLR group (*n* = 182), and the high NLR group (*n* = 22), respectively, comparatively presented with those of the whole study group in Table 1 above.

Dividing the patients into these two groups, we found that there is a homogeneity regarding gender (*p* = 0.579), age of the patients (*p* = 0.128), number of platelets (*p* = 0.142), and biochemical determinations of hemoglobin (*p* = 0.185), total protein (*p* = 0.566), tumoral stage (*p* = 0.060), and white blood count (*p* = 0.108). However, we could notice statistically significant differences in terms of death (*p* = 0.025) and anastomotic leakage (*p* < 0.001), indicating that patients with increased NLR had greater risk of anastomotic leakage as a source of mortality.

We analyzed the appearance of dehiscence in the group with high NLR and with low NLR, respectively, in order to establish the possible connection between the anastomotic leak and the NLR level. We obtained a statistically significant difference (*p* < 0.001) using the Pearson Chi-Square test with an odd ratio of 17.62 for the group of patients with preoperative NLR equal of higher than 3.54.

Comprising of, on the one hand, the subjects with anastomotic leakage and, on the other hand, the patients that presented a good postoperative outcome without complications, their parameters are comparatively presented in Table 3.

Comparing the group of patients who developed anastomotic fistula with those without complications, we found statistically significant differences for NLR values (*p* < 0.001), stage of cancer (*p* = 0.006), and mortality (*p* = 0.049), while age of the patients (*p* = 0.702), gender (*p* = 0.272), platelet count (*p* = 0.148), total serum protein (*p* = 0.225), white blood count (*p* = 0.316), and hemoglobin (*p* = 0.854) did not present any statistically significant differences.

The mean value for NLR in the group of patients with anastomotic leakage was increased to 5.83 ± 1.68 compared to the group that presented a postoperative evolution without complications, in which it was at mean values of 2.44 ± 0.51. The independent t test showed that the differences in mean values for the two groups were statistically significant (*p* < 0.001), demonstrating that patients with anastomotic fistula had higher NLR values.

## 4. Discussion

The relationship between cancer and inflammation was first investigated in 1863, by Virchow, when it was hypothesized that the origin of cancer is from the sites of chronic inflammation, with the presence of leukocytes among neoplastic cells being a regular finding [16,17]. In this context, an inflammatory environment is thought to be essential in the development of most tumors, so systemic inflammation plays a crucial role in cancer occurrence as well as in local and systemic dissemination of neoplastic cells [18].

In the last decade, numerous studies on digestive tumors in different stages have raised the hypothesis that hematological inflammatory markers of the systemic inflammatory response, the most widely used being NLR, may become predictive of survival. Thus, NLR may become a good indicator of systemic inflammatory response [19].

Numerous studies have shown that the natural ratio between neutrophils and lymphocytes is small, reflecting a balance between inflammation and neoangiogenesis on the one hand (which increases the number of neutrophils) and the body’s immune response on the other (reflected by the number of lymphocytes). An increased number of neutrophils is associated with a low survival rate, while an increased number of lymphocytes is associated with an increased rate and better prognosis [20].

Given the above, systemic inflammation and patients’ outcome can be estimated by the NLR, a simple, cheap, reproducible, and widely available parameter, that predicts the survival of cancer patients [21].

Several studies concluded that increased NLR is associated with lower survival in patients with various malignancies, and can predict the prognosis of advanced gastric cancer [22,23,24]. No explanation for a lower survival rate has been identified; however, a higher NLR value is associated with poor nutritional and immune status. More precisely, some affection of the regulation function of natural killer cells, cytotoxic T lymphocytes, and antigen presenting cells can be involved [25,26]. Increased NLR is also associated with the shortening of the time for local recurrence appearance, as well as a lower overall survival. Immune status, which is dependent on leukocytes, is impaired in these patients, which may explain the interdependence between elevated NLR, tumor staging (advanced stage), and poor prognosis [20].

NLR can predict the onset of anastomotic fistula and can assess early mortality. As we are aware, after searching the literature, our study represents the first one to establish anastomotic leakage after gastric resections for cancer in patients presenting high preoperative NLR values.

Anastomosis on the digestive tract results in the creation of a lesion that subjects the epithelium to a healing process that depends on the balance between migration, proliferation, and differentiation of epithelial cells adjacent to the injured area [27]. The process of intestinal scarring is composed of four phases: hemostasis, inflammation, proliferation, and tissue remodeling or resolution (Gosain and DiPietro 2014), phases that must occur in a correct sequence, at a certain time, with a certain duration of time, and with optimal intensity. After performing the anastomosis, neutrophils are the first cells to be attracted to the focus, initiating an inflammatory process that represents the first stage in healing, starting just one hour after the anastomosis has been done and remaining at high levels for 48 h [28]. In the anastomotic trance, neutrophils play the role of phagocyting foreign particles and bacteria. After that, the degranulation process takes place, releasing toxic substances like lactoferin, cathepsin, and proteases. All of these substances will destroy bacteria as well asnecrotic tissue from the area. Recent studies have also found that bacteria in the extracellular space are killed by chromatin and proteases, which are also secreted by neutrophils. Free oxygen radicals are formed in the degradation process of necrotic tissues, foreign particles, and bacteria. Chlorine has the property of linking free radicals, thus resulting in wound sterilization [29].

Neutrophils are attracted to the anastomosis trance, where they become activated and release oxidants and hydrolytic enzymes in the healing process [30]. Activated neutrophils play a direct role in phagocytosis and the clearance of bacteria in the wound, replacing the role of macrophages whose activity is suppressed [31].

The involvement of lymphocytes takes place in a second stage beyond the first 72 h, having a role in modulating the healing processes through the synthesis of the extracellular matrix and the remodeling collagen. There are studies in which the inhibition of T lymphocytes has resulted in decreased healing tissue resistance and poor collagen synthesis [32].

The first stage of healing, the inflammatory one, lasts as long as it takes to phagocytose bacteria and foreign particles from the digestive lumen. Pre-existing proinflammatory conditions will lead to abnormal prolongation of this stage, which results in the transition to the chronic inflammatory phase, with additional tissue injury and delayed synthesis of the extracellular matrix [33]. In our case, this will lead to a deficient cure of anastomosis with dehiscence.

We found a higher mortality in the group of patients with the higher preoperative value of NLR, which is concordant with data from literature. Several recent studies have shown that inflammation associated with malignant tumors promotes local development, tumor progression, and hematological dissemination [34,35]. High NLR values correlate with low prognosis and an advanced stage of cancerous tumors in a variety of malignant breast tumors [36,37], and tumors located in the kidney [38,39], nasopharyngeal [40,41], head, and neck [42].

We divided patients into groups with low NLR value and high NLR value, respectively, thus allowing the most accurate stratification of the patients according to fistula and death risk. The cutoff value of NLR that we found corresponds to other studies from the literature, with regards to postoperative outcome and mortality for patients with gastric malignant tumors [43].

With the data from our study, we wanted to show that NLR values are significantly higher in patients with gastric resections for cancer who have developed anastomotic fistula. In the group with high NLR (greater than or equal to 3.54), the odds ratio (OR) for anastomotic leakage was 17.62. Our data are concordant with other studies [22,44]. Following these observations, we concluded that this preoperative hematological parameter (NLR) can be proposed and used as a predictor of anastomotic leakage in gastric resections for malignant tumors, as it is a useful tool to stratify patients according to risks and helps to set the optimal time for surgical radical intervention.

There is a main limitation for our study that is related to the fact that it was a single-center study. In these conditions, it is difficult to generalize the results. A multicenter study, including a larger number of patients, is needed to draw more solid conclusions.

## 5. Conclusions

An increased preoperative value for NLR is a good negative predictor of anastomotic leakage in patients who need gastric resections for cancer.

NLR was significantly higher in patients who died after radical gastric surgery.

We recommend the preoperative use of NLR as a predictive marker of fistulas in order to customize preoperative assessment and to choose the best surgical technique.

## Figures and Tables

**Table 1:** Characteristics of the groups with high NLR and low NLR, respectively.

	Total, Mean Value ± Standard Deviation (Extreme Values)	High NLR (*n* = 22)	Low NLR (*n* = 182)	*p*-Value
Age	67.75 ± 9.73 (32, 91)	63.50±13.82	68.27 ± 9.03	0.128
Gender %				0.579
Male	128	15	113	
Female	76	7	69	
Fistula				<0.001 *
Yes	22	14	4	
No	182	8	174	
Stage				0.060
I	24	1	23	
II	65	4	61	
III	94	8	86	
IV	21	6	16	
Platelets × 10^3^/μL	268.82 ± 110.17 (51, 611)	236.18 ± 93.88	272.76 ± 111.55	0.142
Leukocites × 10^3^/μL	8.05 ± 2.66 (4.01, 22.13)	9.46 ± 4.35	7.88 ± 2.33	0.108
Hemoglobin g/dL	11.87 ± 2.45 (4.7, 16.63)	11.21 ± 2.26	11.95 ± 2.47	0.185
Serum total proteins g/dL	6.44 ± 0.66 (4.02, 8.10)	6.52 ± 0.58	6.44 ± 0.67	0.566
Death	6	5	1	0.025 *

* *p* less than 0.05 is statistically significant—independent t test with homogeneity of variations in Levene test. NLR: neutrophile/lymfocyte ratio.

**Table 2:** NLR mean values for different tumor stages.

Stage	N	Mean	Std. Deviation	95% Confidence Interval for Mean		Minimum	Maximum
				Lower Bound	Upper Bound		
I	24	2.52	0.94	2.1303	2.9291	1.97	6.72
II	65	2.67	0.90	2.4464	2.8961	2.00	6.39
III	94	2.61	1.01	2.4101	2.8276	1.49	9.75
IV	21	3.45	2.13	2.4784	4.4244	1.61	7.80
Total	204	2.71	1.16	2.5502	2.8713	1.49	9.75

**Table 3:** Variations between the group with anastomotic dehiscence and the group with normal anastomotic healing.

	Anastomotic Dehiscence (*n* = 16)	Normal Anastomotic Healing (*n*= 188)	*p*-Value
Age	64.56 ± 14.90	68.03±9.17	0.702
Gender %			0.272
Male	12	128	
Female	4	60	
NLR	5.83 ± 1.68	2.44 ± 0.51	<0.001 *
Stage			
I	1	23	0.006 *
II	4	60	
III	6	88	
IV	5	17	
Platelets × 10^3^/μL	255.14 ± 105.22	287.98 ± 115.95	0.148
Leukocites × 10^3^/μL	9.06 ± 4.19	7.96 ± 2.48	0.316
Hemoglobin g/dL	11.76 ± 2.40	11.88 ± 2.46	0.854
Total serum proteins g/dL	6.64 ± 0.67	6.43 ± 0.66	0.225
Death	4	2	0.049 *

* *p* less than 0.05 is statistically significant—independent t test with homogeneity of variations in Levene test.
